# Cultural differences in self-reported empathy in Indonesia

**DOI:** 10.1038/s41598-025-16075-5

**Published:** 2025-10-01

**Authors:** Sarah Nila, Christine Webb, Bambang Suryobroto, Alecia Carter

**Affiliations:** 1 Department of Psychology, Durham University, Stockton Rd, Durham, DH1 3LE UK; 2 Department of Anthropology, University College London, 14 Taviton St, London, WC1H 0BW UK; 3 Department of Human Evolutionary Biology, Harvard University, Cambridge, MA USA; 4 Department of Biology, IPB University, Dramaga, Indonesia

**Keywords:** Cross-cultural empathy, Prosociality, Indonesia, Non-WEIRD society, Evolutionary developmental biology, Evolutionary theory

## Abstract

Empathy, comprising cognitive and affective components, is linked to numerous individual and societal benefits, including enhanced well-being and prosocial behaviours. However, empathy might also hinder societal flourishing due to biases resulting from factors like social closeness. This study explored cultural variation in empathy within Indonesia, a non-WEIRD context with diverse ethnicities, religions, and social structures. We hypothesized that cultural factors such as ethnicity, primary residence, religious beliefs, and social class would predict empathy differences. Using standardised questionnaires, we assessed helping behaviour, situational empathic concern, directed empathic concern, and personal distress in 2869 Indonesian adults. Regression analyses revealed that gender, age, ethnicity, residence, religion, and perceived social class significantly predicted empathy components. Notably, Javanese and Sundanese participants reported higher empathy levels than Minangkabau, and Muslims showed more empathy than other religious groups. Additionally, higher perceived social status correlated with increased helping behaviour and emotional empathy, but it also correlated with lower personal distress. These findings highlight the complex interplay of cultural factors in shaping empathy, with implications for understanding socioemotional dynamics across diverse societies.

**Supplementary Information:**

The online version contains supplementary material available at 10.1038/s41598-025-16075-5.

## Introduction

Empathy is the capacity to share and understand the emotions of others, and to respond to them with care^1^. In general, empathy is thought to comprise two main components: cognitive empathy and affective empathy. Cognitive empathy is the ability to recognize and understand another’s emotional states, while affective empathy is the ability to share and vicariously experience those states^2,3^.

Empathy is associated with numerous physical and physiological health benefits^4^, including individual well-being, life satisfaction, and self-esteem. Relatedly, empathy is known to promote positive social interactions and prosocial acts, including helping, donating, and volunteering behaviour^5–9^. For these reasons, among others, empathy is often considered a core component of individual social functioning and success.

Despite these individual benefits, recent research suggests that empathy may actually hinder societal flourishing^10^, in part because it is often biased by social closeness and similarity. For example, Zhao et al.^11^ demonstrated empathy’s in-group bias, where people showed more empathy for those from the same ethnicity (in-group) than for those from a different ethnicity (out-group)^12,13^. Neumann et al.^13^ found that when Caucasian and Asian participants viewed negative photographs (e.g., injury or illness) of both Caucasian and Asian stimuli, participants gave higher empathy ratings for targets of the same ethnicity than for targets of the other ethnicity.

While in-group closeness is a well-known driver of variation in empathy, recent research has highlighted geographic variation in empathy both within and between countries (see Refs.^9,14–18^ for more details), suggesting that broader sociocultural mechanisms may influence empathy’s expression. This is consistent with the more general, widely-held notion that sociocultural factors play a key role in shaping human psychology and behaviour (e.g.,^19,20^). For example, one study involving nearly 80,000 American residential adults found that self-reported cognitive and affective empathy varied considerably across the 50 U.S. states, and correlated positively with prosocial behaviours (e.g., volunteering) and negatively with antisocial behaviours (e.g., robbery) retrieved from public government databases^16^. Additionally, a comparison of empathy across 63 countries showed that people from collectivist countries (those with tightly knit social networks and high interdependence on others) self-reported higher empathy using a standardised questionnaire than those from individualist countries (those with loosely knit social networks and high individuality)^9^. Similarly, Goodwin and Giles^14^ found that, compared with British participants, Indonesian participants were more willing to offer help to support other people (i.e., strangers). Contrary to the results from the studies above, Cassels et al.^15^ and Zheng et al.^18^ found that their Western participants reported higher empathy than Asian participants. Notwithstanding these exceptions, empathy is typically studied at the individual level, and thus relatively less is known about how this socioemotional response varies across cultures.

Although some evidence points to the social transmission of empathy, as predicted by sociocultural frameworks, it is not yet clear which aspects of culture could be responsible for differences in its expression. Some studies speculate that country of origin (e.g.,^15^), home country (e.g.,^21^), ethnicity (e.g.,^22^), or religion (e.g., ^23^) might predict variation in empathy. Another facet of culture that could conceivably predict cross-cultural variation in empathy is social class. Social class and socioeconomic status (SES) capture individual’s position in society. SES can be defined as differential access to desired resources including materials (e.g., income, wealth), humans (e.g., education, skills), and social (e.g., networks, connections) ^24. Social class reflects both material resources^ and perceived social standing in relation to others in society^25,26^. SES is commonly measured using indicators such as income, education, and occupation ^24^. Additionally, respondents can use comparative self-ranking, where individuals assess their position in society concerning others in terms of income, education, and occupational status^25,27^ through the MacArthur Scale, where respondents mark their social position on a picture of a ladder^28^. For example, preschool-aged children already differ in their emotional and motivational empathy: in an experiment to test preschoolers’ empathic responses, children from Western cultures directed more empathic concern towards an adult who was sad, as compared to children from South-East Asian cultures, who were themselves more distressed by the adult’s distress^29^. The authors of this study suggested that the difference in the children’s responses may be due to cultural differences in the hierarchical status of adults, with South-East Asian children showing greater inhibition to approach and offer help to relatively higher-status adults^29^. Such a pattern could be found in adults, where expressed empathy is limited in societies with greater social class. Last but not least, sociocultural insights on empathy may be limited in part due to an emphasis on Western, Educated, Industrialized, Rich, Democratic (WEIRD) societies in psychological research more generally^30^.

Our study aimed to examine aspects of culture that may account for variation in empathy in a non-WEIRD cultural context, with a specific focus on affective empathy—where emotional bonds play a significant role in shaping social interactions. Indonesia offers an ideal setting for exploring cross-cultural differences in empathy, given its rich diversity—over 600 ethnic groups and multiple religious beliefs—unified under a single government and a common national language, Bahasa Indonesia^31,32^. Indonesian society is generally collectivist, having been described as emphasizing cooperation, conformity to authority, harmonious relationships, community involvement, and shared goals^33–36^. This collectivist culture is shaped by strong communal values alongside significant cultural and ethnic diversity. Beyond collectivist norms that foster solidarity and close social ties, other influential factors include religion (with Islam as the majority faith), strong family values, and social norms centred on harmony^37,38^. These elements may meaningfully shape how empathy is perceived and expressed in Indonesia.

We predicted that aspects of culture in Indonesia—ethnicity, place of residence, religious beliefs, and/or social class—would predict differences in empathy. Aside from collecting detailed demographic information, we used a combination of three standardised questionnaires: the MacArthur scale to measure social class or subjective socioeconomic status^39^, the Interpersonal Reactivity Index (IRI) to measure dispositional empathy^40^, and the General Social Survey (GSS) to measure helping behaviour towards strangers and relatives^41^. In the IRI questionnaire, we focused on affective empathy, as it aligns with Indonesia’s emphasis on emotional connectedness and social harmony. In collectivist cultures, shared emotions conceivably play a more significant role in shaping relationships than cognitive perspective-taking. Since our study relied on self-reports, affective empathy was likely more relevant to daily interactions, whereas cognitive empathy requires deeper reflection.

## Results

### Does culture influence self-reported empathy and empathic behaviour?

To examine possible associations between cultural traits and empathy, we conducted four regressions with each empathy factor as a response: helping behaviour (M1), situational empathic concern (M2), directed empathic concern (M3), and personal distress (M4). In M1, gender, age, ethnicity, primary residence, religious belief, and perceived social class each explained variation in helping behaviour to strangers and relatives (Table 1). Men reported significantly more helping than women (*p* < .001). Moreover, older participants reported significantly more empathic behaviour than younger participants (*p* = .002). Ethnicity and region of residence were also significant predictors of helping behaviour. In particular, Javanese participants reported significantly more helping behaviour than Minangkabau participants (*p* = .002). Participants living on Java however reported significantly less helping behaviour than those who lived on Sumatra, Sulawesi, and Other islands (*p* = .019, 0.019, and 0.017, respectively). Muslim people reported significantly more empathic behaviour than Christian, Hindu, Buddhist, and Other belief (all *p* < .001). Finally, participants who perceived themselves as having higher social status reported significantly more helping behaviour towards strangers and relatives than did those who perceived themselves as lower social status (*p* < .001). Additionally, post-hoc pairwise Anova (Table SM3) showed that respondents identifying as Sundanese were significantly different on empathic behaviour from those identifying as Minangkabau and other ethnicity. There was no difference between respondents based on region of residence (Table SM4). Post-hoc pairwise Anova showed that most pair-wise comparisons between religions differed: Islam was significantly different from Christianity, Hinduism, Buddhism, and Others; and Christianity was different from Buddhism and Other (Table SM5).

**Table 1:** Tobit model censored regression of each empathy factors.

	M1		M2		M3		M4		
	β	*p*-value	β	*p*-value	β	*p*-value	β		*p*-value
Intercept	**2.987**	**< 0.001**	**3.274**	**< 0.001**	**3.176**	**< 0.001**	**3.941**		**< 0.001**
Gender (Woman)	**− 0.222**	**< 0.001**	**0.145**	**< 0.001**	**0.462**	**< 0.001**	**0.406**		**< 0.001**
Age	**0.006**	**0.002**	**0.005**	**0.005**	0.002	0.331	− 0.017		**< 0.001**
Ethnic (Minangkabau)	**− 0.166**	**0.002**	**− 0.165**	**0.002**	− 0.014	0.836	**− 0.149**		**0.008**
Ethnic (Others)	0.019	0.609	− 0.001	0.985	**0.141**	**0.004**	− 0.017		0.679
Ethnic (Sundanese)	0.053	0.161	0.020	0.616	0.061	0.240	0.095		0.028
Region of residence (Kalimantan)	0.008	0.919	0.087	0.300	0.024	0.824	− 0.032		0.715
Region of residence (Others)	**0.178**	**0.025**	**0.171**	**0.044**	0.158	0.144	0.106		0.236
Region of residence (Papua)	0.182	0.177	**0.441**	**0.002**	**0.651**	**< 0.001**	0.145		0.340
Region of residence (Sulawesi)	**0.195**	**0.003**	**0.139**	**0.049**	**0.301**	**< 0.001**	− 0.027		0.179
Region of residence (Sumatera)	**0.118**	**0.008**	**0.098**	**0.037**	0.087	0.148	0.045		0.713
Religious beliefs (Christian)	**− 0.335**	**< 0.001**	**− 0.172**	**< 0.001**	− 0.364	**< 0.001**	0.036		0.490
Religious beliefs (Hinduism)	**− 0.614**	**< 0.001**	− 0.204	0.192	**− 0.560**	**0.005**	0.178		0.286
Religious beliefs (Buddhism)	**− 0.785**	**< 0.001**	− 0.142	0.452	− 0.230	0.336	− 0.309		0.119
Religious beliefs (Others)	**− 0.855**	**< 0.001**	− 0.861	**< 0.001**	− 0.7678	**< 0.001**	**− 0.502**		**0.001**
perceived social class	**0.003**	**< 0.001**	0.001	0.159	**0.004**	**0.005**	− 0.007		**< 0.001**

Significant values are in bold.

M1 = Helping behaviour, M2 = Situational EC, M3 = Directed EC, M4 = PD. The reference category for gender was men, ethnicity was Javanese, region of residence was Java, and religious belief was Muslim.

In M2, gender, age, ethnicity, primary residence, and religious beliefs each explained variation in participants’ situational EC (Table 1). Women reported significantly higher situational EC than did men (*p* < .001). Moreover, older participants reported significantly higher situational EC than did younger individuals (*p* = .017). In the full model, ethnicity and primary residence were also significant predictors of situational EC. However, post-hoc pairwise Anova showed no significant difference among ethnicities and among primary residence on the situational EC (Tables SM3–SM4). For religion, the post-hoc pairwise Anova showed significantly difference among religions. Muslim participants reported significantly higher situational EC than Christian, Hindu, Buddhist, and Other belief (Table SM5).

In M3, gender, ethnicity, primary residence, religious beliefs, and perceived social class each explained variation in directed EC (Table 1). Women reported higher directed EC than men (*p* < .001). Ethnicity was also a significant predictor of directed EC, but this was not supported by post-hoc pairwise Anova (Table SM3). Post-hoc pairwise Anova tests showed that region of residence helped explain variation in directed EC: people who lived on Sulawesi Island reported directed EC significantly higher from people who lived on Java and Other islands (Table S4). We also found that Muslim participants had significantly higher directed EC than did participants espousing Christian, Hindu, Buddhist, and Other beliefs in post-hoc pairwise Anova (Table SM5). Finally, participants who perceived themselves as having higher social status reported significantly higher directed EC than did those who perceived themselves as lower social status (*p* = .009).

In M4, gender, age, and perceived social class each explained variation in PD (Table 1). Women reported significantly higher PD than men (*p* < .001). Moreover, younger participants reported higher PD than older individuals (*p* < .001). Finally, participants who perceived themselves as having lower social status reported significantly higher PD than did those who perceived themselves as higher social status (*p* < .001). Additionally, the post-hoc pairwise Anova test (Table S3) showed that Sundanese was significantly higher from Minangkabau. Post-hoc pairwise Anovas revealed no significant differences between any regions on PD (Table SM4). For religion, post-hoc pairwise Anova showed that participants espousing Other religious belief reported significantly lower PD than did Muslim, Christian, and Hindu participants (Table SM5).

## Discussion

This study aimed to examine which aspects of culture might underlie patterns of empathy in Indonesia. In line with previous research, we found four components that described empathy in our self-report sample: helping behaviour, situational EC, directed EC, and PD. While the IRI originally conceptualised PD as self-oriented discomfort when witnessing others’ suffering, our exploratory factor analysis revealed that these items either loaded weakly or were dispersed across other factors. This divergence may be attributed to cultural influences. In collectivist societies like Indonesia, personal distress may be downplayed or moderated by social norms that prioritise group harmony over individual emotional expression^37,38^. As a result, PD in our study does not emerge as a distinct factor but instead appears intertwined with EC—both situational and directed. This suggests that emotional discomfort is more context-dependent rather than purely egocentric. Consistent with our hypothesis, we found that ethnicity, primary residence, religious beliefs, and perceived social class all influenced the components of self-reported empathy that we measured in Indonesians. In addition, we also found consistent effects of the control variables gender and age on self-reported empathy across multiple factors. Before going further, it is important to emphasize that these findings do not imply that any ethnicity, area of residence, religion, or social class is superior or inferior (or inherently more or less empathic), but that these patterns arise through cultural factors that moderate empathy’s expression.

As has been demonstrated previously (^11,14,15,18^ for details), we found that ethnicity was a predictor of variation in empathy. Javanese and Sundanese participants had higher helping behaviour and PD than Minangkabau respondents. Javanese and Sundanese are the two largest ethnic groups in Indonesia and the dominant ethnic groups in Java^42^. As noted in Eisenberg et al.^42^, previous reports from anthropologists, sociologists, and other scholars describe Javanese culture as emphasizing awareness of how one’s actions impact others, discouraging socially disruptive behaviours, and fostering prosocial attitudes. More broadly, traditional Indonesian society—particularly in Java—has been characterized by strong values of cooperation, respect for authority, harmonious relationships, community engagement, and shared goals^33–36^. The Javanese concept of *rukun* (social harmony) fosters cooperation within close-knit communities^43^, while Sundanese social norms promote *silih asah*,* silih asih*,* silih asuh* (mutual teaching, loving, and caring)^44^. In contrast, the Minangkabau exhibit a more individualistic and commercialized orientation^45,46^, which may contribute to their lower reported empathy. More broadly, people from collectivist cultures may exhibit greater empathic concern, as their sense of well-being is often intertwined with others’^9^. However, this interdependence can also influence the balance between maintaining social harmony and addressing individual needs. For instance, prioritizing in-group cohesion may discourage individuals from openly expressing distress, potentially suppressing empathy in certain contexts. Our findings reflect Indonesia’s rich cultural diversity and the nuanced ways in which collectivism shapes empathic behaviour.

To a lesser extent, the place of primary residence also affected self-reported helping behaviour, situational EC, and directed EC. In Indonesia, urban environments—particularly in Java—tend to be more individualistic due to economic migration and weaker kinship ties. Urbanization may reduce social obligations that typically enhance empathy in rural settings, where interdependence is stronger. This aligns with research showing that rural communities foster greater social cohesion and prosocial behaviours. Empathy has been linked to migratory trends and ecological conditions^47^. For example, people who live in smaller communities (e.g., villages) engage in greater empathic behaviour than people who live in larger societies^17,48^. Similarly, more urbanized environments have been associated with reduced social contact among neighbours and a decreased willingness to help and show consideration toward strangers^49–51^. These patterns may help explain why respondents residing in Java reported lower empathic tendencies. Data from Statistics Indonesia (BPS^52^) identifies Java as the most populous and urbanized island in the country, potentially leading to increased anonymity and reduced social interactions. This study highlights that while collectivist cultures often report higher empathy levels, significant variability exists due to geographic and ethnic diversity. For instance, urban residents in Java may display lower empathic behaviours compared to individuals in rural areas, demonstrating how ecological and social contexts shape empathy.

Our results that religious beliefs predict multiple facets of self-reported empathy complements other cross-cultural studies^23^. Indonesia’s religious diversity also contributes to varying expressions of empathy. Islam, the dominant religion, places a strong emphasis on charity (*zakat and sedekah*) and collective responsibility^44^, which may explain why Muslim participants reported higher helping behaviours, situational and directed EC, and PD. In contrast, Christian and Buddhist participants—who are a minority in Indonesia—may navigate different social expectations, possibly influencing how they express empathy towards in-group versus out-group members. In our sample, Muslims reported significantly higher helping behaviour, situational EC, PD, and directed EC. Previous research linking empathy and religion has focussed on religiosity rather than respondents’ (potentially nominal) religion, showing that religious people (who hold religious beliefs) and actively religious people (who pray and/or attend worship in monotheistic religions) reported higher empathy and more helping behaviour^53^. Our study did not ask people about their religiosity, but it could be that in predominantly Muslim Indonesia, where communal prayer is common, Indonesian Muslims have, on average, greater religiosity than members identifying with other religions. Our findings also showed variation in empathy among other religions, aligning with research by Cohen and Varnum^47^, which suggests that religions serve as cultural influences that shape various aspects of psychological functioning. Religious culture can influence work ethics and moral judgments, which may also contribute to variation in empathic responses observed among individuals of different faiths.

Finally, regarding our predictors of interest, we found that perceived social class had a positive correlation with helping behaviour and directed EC, and a negative correlation with PD. These results suggest that those who perceived themselves to be of a higher education and economic status have increased levels of self-reported helping behaviour and emotional empathy. This finding is in accordance with previous studies that socioeconomic position was positively correlated with helping behaviour^54,55^. Korndörfer et al.^54^ suggested that this arises because empathic and helping behaviour are costly, as they consume individuals’ resources, and that individuals from lower status had fewer resources with which to perform this costly behaviour^24,56,57^. Future research could conduct interviews or further surveys to ask respondents about their motivations for and hurdles to helping others to test this further. We found gender differences in helping behaviour, situational EC, PD, and directed EC. Men reported significantly higher helping behaviour than women, whereas women reported higher situational EC, PD, and directed EC than men. In contrast to previous studies^58,59^, Indonesian men reported higher helping behaviour than women. One possible explanation for this finding is a cultural focus on heroic and chivalrous behaviour in men suggested by Eagly and Crowley^60^. We collected data during Covid-19 pandemic in which lockdown was enforced; helping behaviour during this situation could be riskier and thus categorised as heroic behaviour. Another possible explanation is a higher reported helping behaviour by men was influenced by norms and self-presentation in the society^59^. Carlo and Randall^8^ reported that men engage in more public helping or helping other people when they are being observed, which could be the case for helping strangers.

Previous studies have also shown that, as was the case in our study, women reported higher empathy (empathic concern and personal distress) than men^15,40,61–67^). This could be due to the social expectations for gender roles across cultures^21^. Women are socialised and expected to be more interpersonally sensitive and more caring than men^61,68^, particularly in more Western cultures where women are expected to be warm-hearted and openly affectionate, while men are encouraged to be strong-willed and emotionally invulnerable^69,70^. In Indonesia, similar to most WEIRD countries, women are expected to be warm-hearted, whereas men are expected to be strong-willed^71^.

This study also found that helping behaviour and situational EC increased with age, and PD decreased with age. This result is in contrast with a study on Italian individuals by Cavallini et al.^72^ that found age negatively correlated with self-report helping behaviour (also measured via the GSS questionnaire). The authors predicted that this may be due to the age-related decline in physical resources necessary to act prosocially and is in line with a reduction in acts that involve active engagement, such as helping with housework. Despite the older adults’ good generativity (concern for and commitment to the well-being of the next generation), their physical limitations seem to pose obstacles to generative behaviours^73^. Moreover, our finding is in line with Sze et al.^74^ and Beadle et al.^75^, who found that older individuals in the USA gave greater charitable donation and exhibited more helping behaviour than younger individuals in an experiment. In addition, previous studies also reported that IRI-EC was significantly higher for older individuals; however, the ratings for IRI-PD were higher in younger individuals^23,65,67^. According to previous studies, IRI-EC was found to increase continuously in adolescence and adulthood^76^ and PD decreased^77,78^.

Our study has several limitations. Our sample may not be representative of Indonesian society on the whole for various reasons. First, the online questionnaire was only accessible to people living in areas with a good internet connection, and therefore might not have reached smaller cities and more rural areas in Indonesia. Moreover, it is possible that people who are more empathetic and prosocial in general would take the time to fill out an online survey. Second, as Smith et al.^41^ and Einolf et al.^79^ pointed out, self-reported empathy and empathic behaviour are subject to social desirability biases. Respondents may not be able to recall very accurately the amount of helping behaviours they participated in over the past year, and certain participant groups (e.g., women/females) may be more likely to endorse/report their own empathic tendencies (e.g.,^80^). Additionally, given that over 600 languages are spoken in Indonesia, and that some populations do not speak Bahasa as their primary language, the diversity of languages could lead to slight variations in how participants interpret survey questions. For instance, Javanese speakers may perceive a question in Bahasa differently from those whose first language is Bahasa. While we did not collect data on participants’ native languages, acknowledging this variability opens the door for future studies to explore how language nuances could further enhance our understanding of empathy across ethnic groups. Levels of self-reported empathy in the present study were consistent with those provided by Butovskaya and colleagues, ^23^ who also assessed empathy among Indonesian participants during the Covid-19 pandemic. Reports of helping others (relatives, friends, neighbours, etc.) were frequent in media, which might have encouraged and increased helping behaviour during this period.

## Methods

### Sampling

We administered online questionnaires to Indonesian adult residents in February and March 2021. The protocol was approved by UCL Research Ethics Committee (No. 18165/001) and IPB University Research Ethics Committee (No. 299/IT3.KEP MSM-IPB/SK/2020). We confirm that all research was performed in accordance with relevant guidelines/regulations, and informed consent was obtained from all participants. The survey was conducted through the Opinio web-based survey platform and distributed via social media (in particular, WhatsApp, Instagram, and Twitter) using a snowball sampling procedure. The lead researcher (S.N.) recruited individuals in their personal social network and additional participants were obtained from this initial sample. Upon receiving information about the general aim of the study, the type of data to be collected, and data use and anonymity, participants were asked to provide their informed consent. At the beginning of the survey, each participant was asked to provide demographic information (gender; age in years); their primary residence (name of islands in Indonesia); as well as their cultural background including their own, their parents’, and their grandparents’ ethnic group; and their religious beliefs. The ethnic groups of participants were confirmed by the ethnicity of their parents and grandparents. All the questions in the survey were translated from English into Bahasa Indonesia by S.N. An independent bilingual translator, unfamiliar with the original items, conducted a back-translation into English. A limited number of discrepancies in meaning were identified, and those that were present were resolved through a process of consensus. All items in the Indonesian version of the questionnaires have been validated through expert judgment. A pilot survey was conducted with 5 participants to assess the clarity, cultural relevance, and accessibility of the translated questionnaire. During the pilot test, none of participant indicated any issues with the questions.

**Fig. 1:**
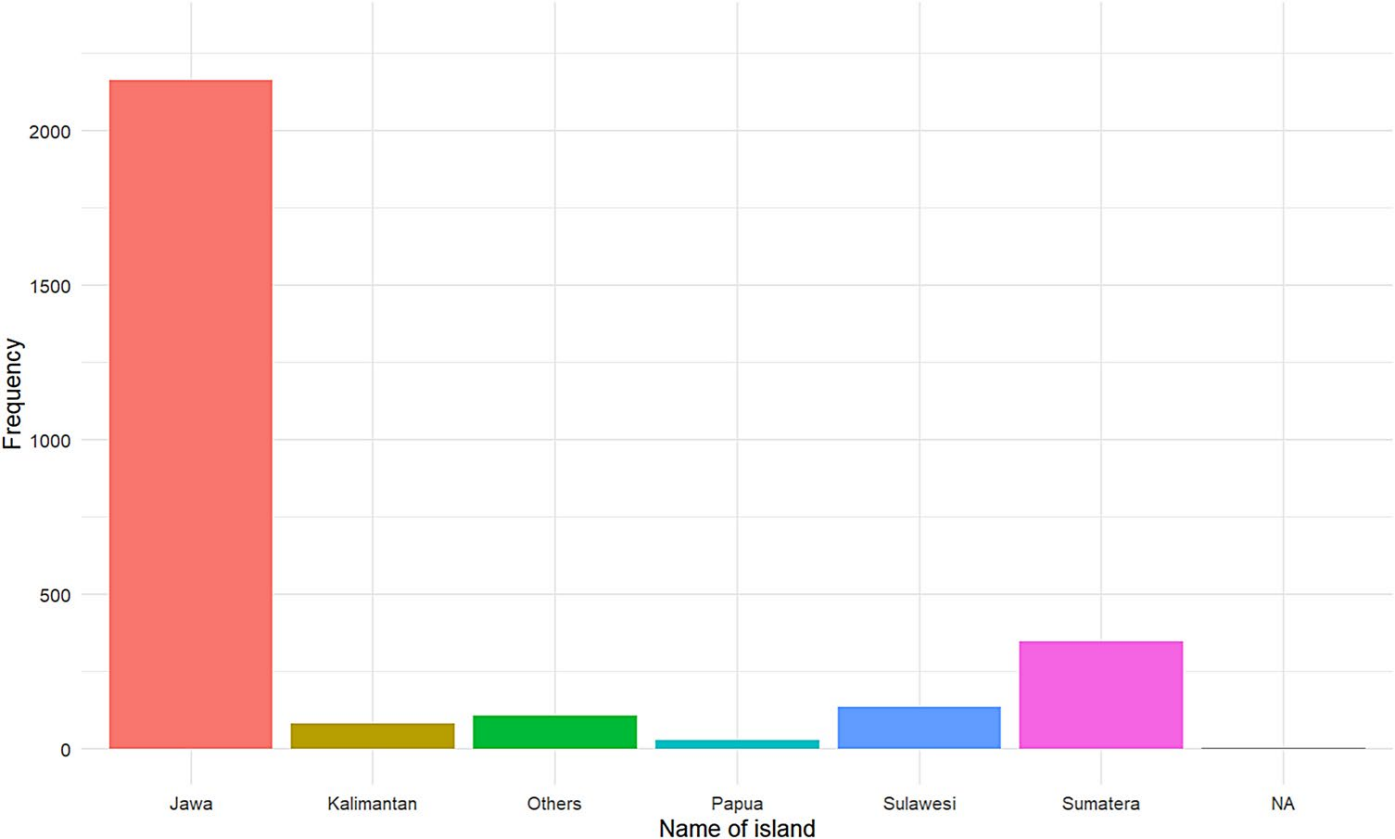
Proportion of participants by island of residence.

In total, 2869 participants responded to the survey (N_male_ = 879, N_female_ = 1990). Participants’ ages ranged from 18 to 70 years (median = 28 ± 8.2 years; men: 29 ± 8.1 years; women: 28 ± 8.2 years). The majority of participants were from Java (75.4%), the most populous island in Indonesia, with other participants from Sumatera (12.1%), Kalimantan (2.9%), Sulawesi (4.7%), Papua (1.0%), and other (3.7%) islands. Additionally, 0.2% of participants did not provide an answer or were categorised as NA (Fig. 1). Approximately one third of participants (32.21%) reported living in a different city than their father whereas the remaining two thirds (67.79%) reported living in the same city. In line with the predominant ethnic group in Indonesia (32; 81), the largest ethnic group to respond were Javanese (42.8%), followed by Sundanese (16.6%), and Minangkabau (9.0%), with the rest being individuals from other ethnic groups (31.1%), or of unknown ethnicity (‘not applicable’ response, 0.6%). Likewise, reflecting the distribution of religions across Indonesia, the majority of respondents were Muslim (85.9%), followed by Christian (11.3%), Hindu (1%), Buddhist (0.6%), and 0.9% of respondents were categorised as following other religious beliefs (Atheist, Agnostic, Sunda wiwitan and Kejawen). Only 0.3% of respondents did not report a religious belief.

## Measures

### Social class

To measure social class, we modified the MacArthur Scale as follows: each participant was asked to compare their socio-economic status (in terms of income, educational level, and occupation) to that of their Indonesian acquaintances (e.g., friends, family, and work group) using a continuous scale (0 on the scale indicated that the participant perceives that they have the least amount of money, poorest education, and least respected job relative to their acquaintances, and 100 on the scale would be the converse, i.e., they perceive themselves to be the richest and most educated with a respected job). Thus, higher numbers were indicative of a higher perceived relative position on the social hierarchy.

Overall, participants reported a higher-than-average social status (*M* = 68.99) compared to their acquaintances. Specifically, one third of participants (34.9%) reported scale values between 75 and 100, 45.8% participants reported scale values between 51 and 74, 12.6% reported a scale value of 50, 5.4% reported scale values 25–49, and 1.3% reported scale values between 0 and 24.

### Emotional empathy

We administered a subset of questions from the Interpersonal Reactive Index (IRI^40^), one of the most widely used questionnaires to measure affective empathy. Specifically, we used the Empathic Concern (EC) subscale (the tendency to experience feelings warmth, sympathy, and concern toward others), and the Personal Distress (PD) subscale (the tendency to have feelings of discomfort and concern when witnessing others’ negative experiences). Each item was rated on a 5-point Likert scale ranging from 0 (does not describe me well) to 4 (describes me very well)^40^. The English and Indonesian versions of the IRI items used in the study appear in Supplementary Material (Table SM1).

Participants’ mean level of self-reported emotional empathy was 3.10 (SD = 0.50). Specifically, the mean level of empathic concern (EC) was 3.24 (SD = 1.51) and 2.97 (SD = 1.41) for personal distress (PD).

### Helping behaviour

Helping behaviour was measured using the 2002 General Social Survey (GSS^41,79^), modified for Indonesia. The 2002 GSS measures 15 different helping behaviours, such as giving food or money to a homeless person, giving directions to a stranger, and donating food to relatives. We removed one item (blood donation) because this practice is not common to all regions in Indonesia, leaving a total of 14 behaviours. The GSS asks individuals to recall how often they had performed a given activity in the past year (see Table 3 for frequency categories). Following Smith^41^ and Einolf et al.^79^. , we transformed the answer into an interval scale representing the annual rate that the behaviour was performed (Table 2). The English and Indonesian versions of all of the GSS items used in the present study appear in Supplementary Material (Table SM2). The proportion of participants who performed each of 14 helping behaviours at least once during the past year is presented in Table 3.

**Table 2:** The categories of frequency with which participants performed GSS helping behaviours and the interval scale for the annual rate.

Frequency	Annual rate
More than once a week	75
Once a week	52
Once a month	12
Two or three times in the past year	2.5
Once in the past year	1
Not at all during the past year	0

**Table 3:** Descriptive statistic for empathic behaviours.

Questions	Percentage having done at least once	Median times/year	Mean times/year	Standard deviation
1. Given food or money to a homeless person	81.4	2–3 (in the last year)	17.7	25.2
2. Carried a stranger’s belongings, like groceries, a suitcase, or shopping bag	60.8	1	9.9	21.2
3. Given direction to a stranger	87.0	2–3	11.9	21.6
4. Gave up your seat for a stranger	78.3	2–3	15.2	25.4
5. Lent an item	52.1	1	6.5	17.2
6. Went out of your way to be kind to a stranger, e.g., by paying a verbal compliment	92.4	12	25.2	29.9
7. Helped someone outside of your household with housework or shopping	81.4	2–3	24.0	30.4
8. Lent a money	88.0	2–3	14.5	23.3
9. Donated food	94.6	12	22.6	27.1
10. Spent time talking with someone who was a bit down or depressed	92.8	12	27.7	30.0
11. Helped somebody to find a job	61.4	1	6.5	16.9

### Statistical analyses

**Fig. 2:**
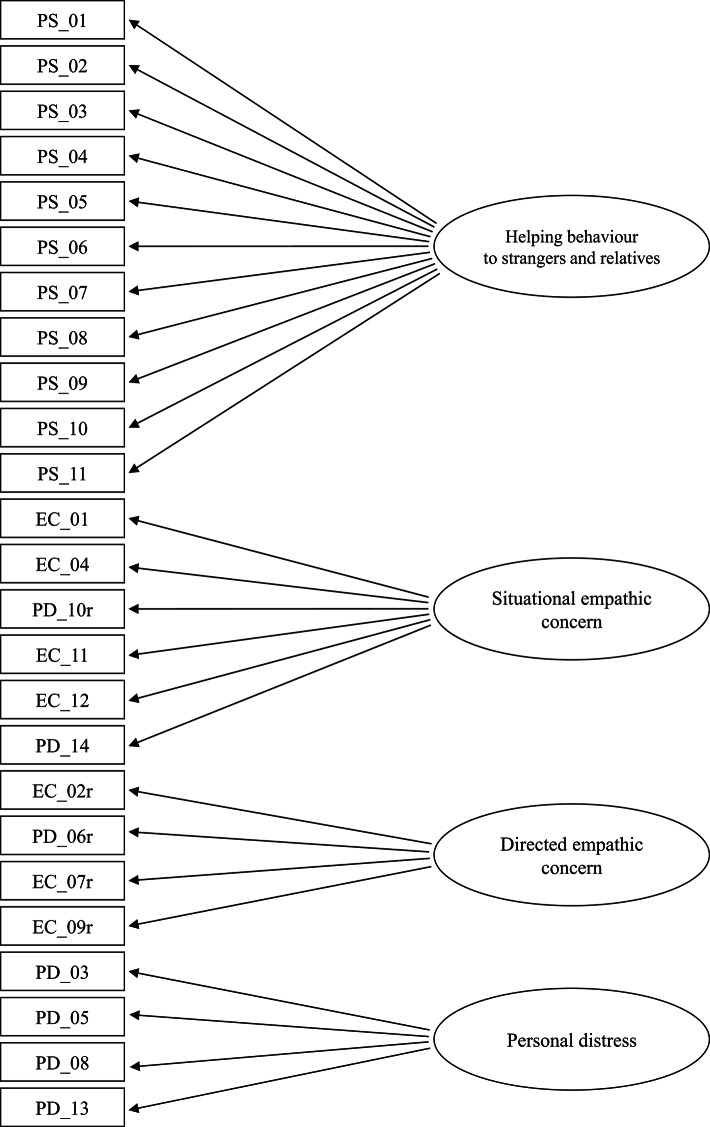
Four-factor of the IRI and 2002 GSS with factor structure identified by item numbers and factor intercorrelations. PS = Helping behaviour to strangers and relatives; EC = Empathic concern; PD = Personal distress; r means reverse score.

Our analyses proceeded in two steps. First, we used a varimax rotated exploratory factor analysis (package psych and GPArotation) to examine the overall structure of empathy in Indonesians from the IRI and the GSS variables (Fig. 2). Four factors were extracted that accounted for 65% of the overall variance, with RMSEA index of 0.039 and fit based upon off diagonal values was 0.98. We labelled these factors: Helping behaviour to strangers and relatives (Factor 1)—encompassing various actions individuals take to assist others, Situational empathic concern (Factor 2)—reflecting empathic sensitivity and responsiveness to others’ distress, encompassing both compassionate concern for those in need and emotional reactivity to distressing situations, Directed empathic concern (Factor 3)—capturing emotional detachment or low empathic concern, indicating a reduced tendency to experience sympathy or distress in response to others’ suffering, and Personal distress (Factor 4)—reflecting a self-oriented emotional reaction to others’ distress, particularly in high-stress or emergency situations. For interpretation purposes, as shown in Table 4, items with a factor loading at least 0.40 were considered to load on that factor (except for 1 item from Factor 2 subscale that had a loading of 0.38). All of the items making up helping behaviour subscale loaded positively on Factor 1. All 5 of the situational empathic concern items loaded positively on Factor 2 (1 item loaded negatively on this factor). The 4 items from the directed empathic concern subscale loaded positively on Factor 3. The 4 items from personal distress loaded positively on Factor 4. To further examine the internal consistency of the subscale of the GSS and IRI, the corrected item-total correlations for each subscale were examined. These correlations ranged from 0.65 to 0.91 for Factor 1, 0.38 to 0.83 for Factor 2, 0.30 to 0.80 for Factor 3, and 0.47 to 0.86 for Factor 4.

**Table 4:** Varimax rotated factor loadings for the GSS and IRI variables.

	F1	F2	F3	F4
PS_01	**0.46**	0.16	0.08	− 0.15
PS_02	**0.63**	0.12	0.00	− 0.08
PS_03	**0.64**	0.07	− 0.01	− 0.05
PS_04	**0.63**	0.05	0.02	− 0.02
PS_05	**0.55**	0.05	0.01	− 0.02
PS_06	**0.60**	0.13	0.03	− 0.05
PS_07	**0.49**	0.07	0.04	0.03
PS_08	**0.54**	0.03	0.04	0.01
PS_09	**0.57**	0.09	0.04	− 0.03
PS_10	**0.50**	0.07	0.07	0.02
PS_11	**0.50**	0.12	− 0.07	− 0.11
EC_01	0.10	**0.47**	0.22	0.07
EC_04	0.15	**0.41**	0.14	0.06
PD_10r	− 0.17	**− 0.39**	0.08	0.27
EC_11	0.10	**0.64**	0.16	0.14
EC_12	0.11	**0.55**	0.10	0.06
PD_14	0.11	**0.61**	0.14	0.14
EC_02r	0.04	0.17	**0.47**	− 0.03
PD_06r	0.02	0.06	**0.59**	0.06
EC_07r	0.05	0.17	**0.61**	− 0.04
EC_09r	0.01	0.11	**0.54**	− 0.03
PD_03	− 0.06	0.09	0.03	**0.64**
PD_05	− 0.12	0.13	0.00	**0.63**
PD_08	− 0.03	0.08	0.00	**0.63**
PD_13	− 0.01	0.02	− 0.08	**0.61**

Significant values are in bold.

Second, to determine which aspects of participants’ cultural background might predict their empathy and helping behaviour, we ran four censored Tobit models (package censReg), one for each factor of the factor analysis as the response variable. The censored Tobit model was used since the response variable data were collected using Likert-type scale and thus have a bounded distribution, but scores were subject to both left and right censoring. As predictor variables, we included participants’ ethnicity, region of residence, religious beliefs, and perceived social class. We also included participants’ age and gender as predictor variables, given prior research showing that females subjectively report more helping behaviour and empathic concern than males (see^82^ for a review) as well as age-related changes in this socioemotional response (see Ref.^83^ for a review). If a multi-factorial predictor variable was significant in the full model, we ran post-hoc pairwise Anova tests to determine the differences between each pair of factors. All analyses were conducted in R version 4.1.1^84^.

## Supplementary Information

Below is the link to the electronic supplementary material.

Supplementary Material 1

## Data Availability

The data used for this paper are available in OSF and accessible using the following link https://osf.io/8xzvb/?view_only=7c6246615b6e4ec4b520e79046ef24a3.
