# Supplementary material for: Cultural differences in self-reported empathy in Indonesia
Source: Sci Rep. 2025 Oct 1;15:34201. doi: 10.1038/s41598-025-16075-5 (PMC12488945; doi:10.1038/s41598-025-16075-5)
Supplement: Supplementary file 1 — Supplementary Material 1 [file 41598_2025_16075_MOESM1_ESM.docx]

SUPPLEMENTARY MATERIALS

**Cultural differences in self-reported empathy in Indonesia**

Sarah NILA, Christine WEBB, Bambang SURYOBROTO, & Alecia CARTER

**Table SM1 Items of the English and Indonesian versions of the Interpersonal Reactivity Index.**

| Items | | |
| --- | --- | --- |
| 1 | I often have tender, concerned feelings for people less fortunate than me  (Saya sering merasa kasihan dan prihatin terhadap orang-orang yang tidak seberuntung saya) | EC |
| 2 | Sometimes I don’t feel very sorry for other people when they are having problems  (Terkadang saya tidak merasa kasihan terhadap orang yang sedang memiliki masalah) | EC* |
| 3 | In emergency situations, I feel apprehensive and ill-at-ease  (Pada situasi darurat, saya merasa gelisah dan tidak nyaman) | PD |
| 4 | When I see someone being taken advantage of, I feel protective towards them  (Ketika saya melihat seseorang sedang dimanfaatkan oleh orang lain, saya merasa perlu melindungi orang tersebut) | EC |
| 5 | I sometimes feel helpless when I am in the middle of a very emotional situation  (Terkadang saya merasa tidak berdaya ketika saya sedang berada di situasi yang sangat emosional) | PD |
| 6 | When I see someone get hurt, I tend to remain calm  (Ketika saya melihat seseorang tersakiti, saya cenderung tetap tenang) | PD* |
| 7 | Other people’s misfortunates do not usually disturb me a great deal  (Kemalangan orang lain biasanya tidak mengganggu saya) | EC* |
| 8 | Being in a tense emotional situation scares me  (Saya takut berada dalam situasi yang menegangkan) | PD |
| 9 | When I see someone being treated unfairly, I sometimes don’t feel very much pity for them  (Saya terkadang tidak merasa kasihan Ketika melihat seseorang diperlakukan tidak adil) | EC* |
| 10 | I am usually pretty effective in dealing with emergencies  (Saya biasanya cukup efektif dalam menangani keadaan darurat) | PD* |
| 11 | I am often quite touched by things that I see happen  (Saya sering merasa tersentuh dengan hal-hal yang saya lihat di sekitar saya) | EC |
| 12 | I would describe myself as a pretty soft-hearted person  (Saya mendeskripsikan diri saya sebagai orang yang berhati lembut) | EC |
| 13 | I tend to lose control during emergencies  (Saya cenderung akan kehilangan control diri saat situasi darurat) | PD |
| 14 | When I see someone who badly needs help in an emergency, I go to pieces  (Saya akan merasa sangat tersentuh ketika melihat seseorang sangat membutuhkan bantuan di kondisi darurat) | PD |

Note. The asterisk sign (*) indicates reversed items. EC = Empathic concern; PD = Personal dostress.

**Table SM2 Items of the English and Indonesian versions of the 2002 GSS.**

| Items | |
| --- | --- |
| 1 | Given food or money to a homeless person  (memberi makan atau uang kepada tunawisma) |
| 2 | Carried a stranger’s belongings, like groceries, a suitcase, or shopping bag  (Membantu membawakan barang milik orang lain (yang tidak dikenal), seperti membawakan bahan makanan, koper, atau tas belanjaan) |
| 3 | Given directions to a stranger  (Membantu menujukkan arah/jalan ketika ada orang tersasar) |
| 4 | Gave up your seat for a stranger  (Memberikan tempat duduk kepada orang lain yang tidak dikenal) |
| 5 | Lent an item  (Meminjamkan sebuah barang kepada orang lain yang tidak dikenal) |
| 6 | Went out of your way to be kind to a stranger, e.g., by paying averbal compliment  (Berbuat baik kepada orang lain yang tidak dikenal, misalnya memberi pujian kepada orang tersebut) |
| 7 | Helped someone outside of your household with housework or shopping  (Membantu orang selain keluarga inti Anda untuk mengerjakan pekerjaan rumah tangga atau belanja) |
| 8 | Lent money  (Meminjamkan uang) |
| 9 | Donated food  (Menyumbang/ mengirimkan makanan) |
| 10 | Spent time talking with someone who was a bit down or depressed  (Menyediakan waktu untuk mengobrol dengan seseorang yang sedang ada masalah atau depresi) |
| 11 | Helped somebody to find a job  (Membantu seseorang mendapatkan pekerjaan) |

Note. The asterisk sign (*) indicates reversed items. EC = Empathic concern; PD = Personal dostress.

**Table SM3 The estimate and p-value adjusted (in brackets) from post-hoc pairwise Anova for family ethnicity.**

|  | M1 | M2 | M3 | M4 |
| --- | --- | --- | --- | --- |
| Minangkabau - Javanese | -0.075 (.111) | -0.075 (.203) | 0.041 (.804) | -0.075 (.342) |
| Others - Javanese | -0.035 (.386) | -0.007 (.992) | 0.052 (.306) | 0.001 (.999) |
| Sundanese - Javanese | 0.059 (.120) | 0.014 (.966) | 0.049 (.527) | 0.068 (.187) |
| Others - Minangkabau | 0.041 (.642) | 0.067 (.316) | 0.010 (.996) | 0.073 (.350) |
| Sundanese - Minangkabau | **0.134 (.002)** | 0.089 (.166) | 0.008 (.999) | **0.139 (.021)** |
| Sundanese - Others | **0.094 (.005)** | 0.021 (.909) | -0.002 (.999) | 0.067 (.248) |

**Table SM4 The estimate and p-value adjusted (in brackets) from post-hoc pairwise Anova for family region of residence.**

|  | M1 | M2 | M3 | M4 |
| --- | --- | --- | --- | --- |
| Kalimantan – Java | -0.039 (.984) | 0.077 (.843) | 0.039 (.996) | -0.061 (.961) |
| Others – Java | -0.016 (.999) | 0.009 (.999) | -0.072 (.899) | 0.012 (.999) |
| Papua – Java | -0.045 (.997) | 0.213 (.359) | 0.288 (.229) | 0.040 (.999) |
| Sulawesi – Java | 0.078 (.476) | 0.063 (.805) | **0.198 (.016)** | -0.088 (.615) |
| Sumatra – Java | 0.020 (.981) | 0.026 (.969) | 0.036 (.939) | -0.006 (.999) |
| Others – Kalimantan | 0.023 (.999) | -0.067 (.969) | -0.111 (.884) | 0.073 (.973) |
| Papua - Kalimantan | -0.007 (.999) | 0.136 (.886) | 0.249 (.559) | 0.101 (.979) |
| Sulawesi – Kalimantan | 0.117 (.557) | -0.014 (.999) | 0.159 (.565) | -0.028 (.999) |
| Sumatra – Kalimantan | 0.059 (.933) | -0.051 (.979) | -0.003 (.999) | 0.054 (.983) |
| Papua – Others | -0.029 (.999) | 0.203 (.546) | 0.360 (.131) | 0.028 (.999) |
| Sulawesi – Others | 0.094 (.696) | 0.053 (.979) | **0.269 (.028)** | -0.100 (.830) |
| Sumatra – Others | 0.036 (.987) | 0.016 (.999) | 0.108 (.712) | -0.019 (.999) |
| Sulawesi – Papua | 0.123 (.841) | -0.150 (.800) | -0.091 (.988) | -0.128 (.927) |
| Sumatra – Papua | 0.066 (.985) | -0.187 (.547) | -0.252 (.416) | -0.046 (.999) |
| Sumatra - Sulawesi | -0.058 (.859) | -0.037 (.987) | -0.161 (.174) | 0.082 (.797) |

**Table SM5 The estimate and p-value adjusted (in brackets) from post-hoc pairwise Anova for family religion.**

|  | M1 | M2 | M3 | M4 |
| --- | --- | --- | --- | --- |
| Christianity – Buddhism | **0.361 (.038)** | **0.045 (<.001)** | **-0.179 (<.001)** | -0.014 (.999) |
| Hinduism – Buddhism | 0.239 (.504) | **0.015 (<.001)** | **-0.303 (<.001)** | 0.132 (.967) |
| Islam – Buddhism | **0.554 (.001)** | **0.128 (<.001)** | **0.011 (<.001)** | -0.016 (.999) |
| Others – Buddhism | 0.062 (.994) | **-0.525 (<.001)** | **-0.506 (<.001)** | -0.470 (.159) |
| Hinduism – Christian | -0.121 (.677) | **-0.029 (<.001)** | **-0.124 (<.001)** | 0.146 (.761) |
| Islam – Christianity | **0.194 (<.001)** | **0.084 (<.001)** | **0.189 (<.001)** | -0.002 (.999) |
| Others – Christianity | **-0.299 (.026)** | **-0.569 (<.001)** | **-0.327 (<.001)** | **-0.456 (.005)** |
| Islam – Hinduism | **0.315 (.005)** | **0.113 (<.001)** | **0.314 (<.001)** | -0.148 (.725) |
| Others – Hinduism | -0.177 (.647) | **-0.541 (<.001)** | **-0.203 (<.001)** | **-0.602 (.005)** |
| Others – Islam | -0.492 **(<.001)** | **-0.654 (<.001)** | **-0.517 (<.001)** | **-0.454 (.004)** |
